# Isolating and characterizing cluster AB Mycobacteriophage NoShow, which encodes lysis proteins shared with cluster H2 phages

**DOI:** 10.1128/MRA.00649-23

**Published:** 2023-09-25

**Authors:** Danielle J. Niblock, Anne R. Winkler, Caroline E. Curtin, Jacqui F. Kokinda, Walter A. Danker, Ignacio Llorente Fernandez, Ava R. Smith, Asad Ali, Gabrielle G. Cruz, Leya C. Givvines, Julia Y. Lee-Soety

**Affiliations:** 1 Department of Biology, Saint Joseph’s University, Philadelphia, Pennsylvania, USA; Department of Biology, Queens College, Queens, New York, USA

**Keywords:** mycobacteriophage, NoShow, cluster AB, lysis

## Abstract

We present here Mycobacteriophage NoShow, isolated from a soil sample collected on the Maguire Campus of Saint Joseph’s University in Merion Station, Pennsylvania. Even though NoShow’s 52,825 bp genome is most similar to phages in cluster AB, its *lysA* and *lysB* genes are most similar to phages in cluster H2.

## ANNOUNCEMENT

**Fig 1:**
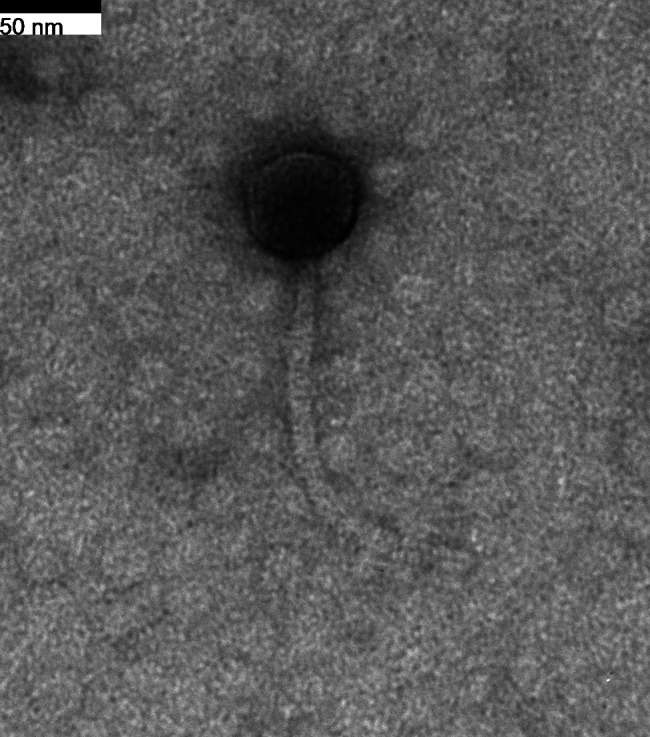
Mycobacteriophage NoShow is a siphovirus. Phage lysates were stained with 1% uranyl acetate. A single phage with an icosahedral capsid head measured 55 nM, and the long, flexible tail was 170 nM.

Bacteriophages have the ability to lyse their bacterial hosts, and uncovering a diversity of phages is of significant clinical value in developing therapeutics to control pathogenic bacteria such as multidrug-resistant *Mycobacterium abscessus* (1–5). Here, we report of phage NoShow, which was isolated from a moist and compacted soil sample collected in January 2021 (39.996504 N and 75.245769 W). The soil was mixed with 7H9 liquid medium, shaken at 37°C for 2 h, and then filtered (0.2 μm pore size). The filtrate was inoculated with *Mycobacterium smegmatis* mc^2^155, incubated with shaking for 2 days at 37°C, filtered, and the filtrate plated in top agar with *M. smegmatis*. NoShow formed clear plaques ~1 mm in diameter after 48 h at 37°C and was purified through multiple rounds of plating. Negatively stained transmission electron microscopy (1% uranyl acetate) revealed a siphovirus morphology (Fig. 1).

NoShow’s DNA was extracted by Phenol-Chloroform (https://phagesdb.org/protocols/88/), prepared for sequencing using the NEB Ultra II library kit, and sequenced on Illumina MiSeq (v3 reagents), yielding 59,426 single-end 150-base reads. Raw reads were assembled and checked for completeness using Newbler (v2.9) and Consed (v29) with default settings (6), respectively, generating single major contigs with 154-fold coverage. NoShow was sorted into cluster AB based on clustering parameters of >35% shared gene content to phages within the Actinobacteriophage database, accessed in August 2021 (7). Within cluster AB, NoShow shares the highest gene content with lytic Mycobacteriophages JacoRen57 (60.8%, Accession #MK279840), Muddy (54.1%, #KF024728), and FF47 (54.0%, #JX901189) (Geneious Prime (2022.0.2)) and has the highest G + C content (60.5%; cluster average of 58.6%). NoShow possessed 11 bp 3′ single-stranded genome termini (5′-CGGAGAGGCTT-3′) (6).

The genome was annotated using PECAAN (v2021) (8), Phamerator (v473) (9), Starterator (v488) (http://phages.wustl.edu/starterator), ARAGORN (v1.2.38) and tRNAscan-SE (v1.3) (10, 11), HHPred [v3.2; PDB_mmCIF70, SCOPe70, Pfam-A, NCBI_Concerved_Domains (CD)] (12), and BlastP [v2.12; Actinobacteriophage Proteins, non-redundant protein sequences (nr)] (13), and then formatted on DNA Master (v5.23.2) (14). Default parameters were used for all software. From NoShow’s 52,825 bp genome, 88 genes were identified but no tRNAs.

Consistent with cluster AB phages, NoShow lacks any identifiable immunity repressor or integrase functions and is, therefore, considered a lytic phage (15–18). Unlike other Cluster AB phages with shared Lysin A and Lysin B protein phamilies (phams), NoShow encodes lysis gene products that are shared with phages from other clusters, including those isolated on different bacterial hosts [protein-encoding genes annotated by PECAAN (v2021) and similarities identified by BlastP (v2.2.26, Actinobacteriophage Proteins as of 4–29-2023 (19))]. For example, NoShow Lysin B is shared with phages in cluster H2, whereas NoShow Lysin A is found in cluster H2 phages and phages isolated on Gordonia spp. NoShow’s Lysin A consists of conserved catalytic amidase and LGFP repeat domains for hydrolyzing peptidoglycan and binding to bacterial cell walls, respectively (20, 21). No conserved protein domains were detected in NoShow’s Lysin B, which is expected to function as a mycolylarabinogalactan esterase (21–23). Holin, a third component of the lysis cassette and involved in the timing of lysis, could not be identified in NoShow (24, 25).

## Data Availability

NoShow is available at GenBank with Accession No. ON108645 and Sequence Read Archive (SRA) No. SRX14483225.
